# Durable Lithium/Selenium Batteries Enabled by the Integration of MOF-Derived Porous Carbon and Alucone Coating

**DOI:** 10.3390/nano11081976

**Published:** 2021-07-31

**Authors:** Mohammad Hossein Aboonasr Shiraz, Erwin Rehl, Hossein Kazemian, Jian Liu

**Affiliations:** 1 School of Engineering, Faculty of Applied Science, The University of British Columbia, Kelowna, BC V1V 1V7, Canada; mohammad.shiraz@ubc.ca; 2 Northern Analytical Lab Services (NALS), University of Northern British Columbia, 3333 University Way, Prince George, BC V2N 4Z9, Canada; Erwin.Rehl@unbc.ca

**Keywords:** metal-organic framework (MOF), zeolitic imidazolate framework (ZIF), zelithium-selenium batteries, alucone, molecular layer deposition, energy storage

## Abstract

Lithium-selenium (Li-Se) batteries are a promising energy storage system in electric vehicles due to their high capacity and good kinetics. However, the shuttle effect issue, caused by polyselenide dissolution from the Se cathode, has hampered the development of Li-Se batteries. Herein, we developed a facile preparation of porous carbon from a metal-organic framework (MOF) to confine Se (Se/CZIF) and protect the Se/CZIF composite with an alucone coating by molecular layer deposition (MLD). The optimal alucone coated Se/CZIF cathode prepared exhibits a one-step reversible charge/discharge process in the carbonate electrolytes. The inhibition of polyselenide dissolution is credited with the improved electrochemical performance, formation of thin and stable solid electrolyte interphase (SEI) layers, and a reduction in charge transfer resistance, thus improving the overall performance of Li-Se batteries.

## 1. Introduction

The rising level of greenhouse gases caused by the increased use of fossil fuels is one of the foremost drivers of climate change. As a result, electric vehicles (EVs) are gaining worldwide popularity as cleaner alternatives to the internal combustion engine. Moreover, many other applications, such as portable devices and stationary and wireless sensors, use lithium-ion batteries (LIBs) to store and supply electricity. However, since lithium-ion batteries using intercalation chemistry are nearing their theoretical limit, it is imperative that a new generation of batteries with greater storage capacity be developed [1,2].

Lithium-sulfur (Li-S) and lithium-selenium (Li-Se) batteries have attracted considerable attention as alternatives to LIBs for EV applications. Li-S batteries provide a high theoretical energy density (1672 mAh g^−1^) but face a major challenge in achieving areal capacities and good durability for practical application due to the poor electronic conductivity of sulfur (5 × 10^−28^ S m^−1^) [3]. On the other hand, Li-Se batteries have the advantage of high electronic conductivity of Se (1 × 10^−3^ S m^−1^) and a slightly lower volumetric capacity of 3254 mAh cm^−3^ to that of sulfur (3467 mAh cm^−3^) [4].

Confining selenium into the porous carbon as a cathode has been demonstrated as a practical approach to increasing Li-Se batteries’ performance [5,6,7]. The impregnation of Se into tiny carbon pores could increase the electronic conductivity, reduce the diffusion length of lithium ions, and enhance the electrochemical properties of Li-Se batteries. It might also prevent the shuttle effect in Se-based batteries caused by polyselenide dissolution from Se cathodes into the electrolyte [7,8,9,10].

Different approaches [6,11,12,13,14,15,16,17] have been investigated to suppress the shuttle effect in Li-Se batteries. One-dimensional porous carbon materials, for example, are extremely desired due to their distinct structural benefits in providing the minimum requirements of a carbon host and an Se space confinement strategy [18,19]. Among various types of carbon preparation, such as pyrolyzed polyacrylonitrile [20,21,22], carbon spheres [23,24,25], and mesoporous carbon nanofibers [19,26], MOF-derived porous carbon piqued the interest of researchers due to its narrow pore size distribution and large pore volume, both of which are desired properties of carbon hosts for Se cathodes [27]. The MOF structure comprises metal ions that are linked by organic ligands. They show high porosity and surface area, with desirable conductivity and thermal/mechanical stability [28]. Therefore, pyrolysis of MOFs could yield porous carbon with all the ideal characteristics needed to obtain high-performance Li-Se batteries.

Molecular layer deposition (MLD) has been explored as a practical ultrathin surface coating approach to suppress the shuttle effect problem in Li-S batteries [29]. The alternative exposure of precursor vapors to a substrate surface in MLD will lead to the deposition of organic/inorganic hybrid films on the targeted sample’s surface [30,31]. For alucone coating, these exposures would be between glycerol (GL) and trimethylaluminum (TMA) to make a conformable thin film. The general steps of the MLD procedure are to (1) supply a dose of TMA vapor, (2) purge to remove oversupplied TMA and any by-products, (3) supply a dose of GL vapor, and (4) purge to remove oversupplied GL and any by-products [30,31,32]. The use of atomic layer deposition (ALD) of Al_2_O_3_ coating for selenium cathodes has been reported in the literature [33]. The beneficial characteristics of alucone MLD coating, such as desirable flexible mechanical properties, universal application, and exhibiting solid and prolonged protection for electrodes during cycling, have attracted researchers investigating this coating for different battery applications [34,35].

Herein, we report a KOH activation-free porous carbon derived from ZIF-8 (a subclass of MOFs) to confine Se in melting-diffusion progress. In addition, further cathode enhancement was applied by alucone MLD coating to suppress the dissolution of polyselenides into the electrolyte, enabling the high usage of active metal and long-lasting cycling performance.

## 2. Materials and Methods

### 2.1. Synthesis of ZIF-8 and Porous Carbon

Zeolitic imidazole framework-8 (ZIF-8) was synthesized using an aqueous solution method [36,37,38]. In a typical process, two different solutions were prepared. The first solution (A) consisted of 1.188 g (3 mmol) of Zn (NO_3_)_2_·6H_2_O dissolved in 6 mL of deionized (DI) water and mixed at room temperature. The second solution (B) was prepared by dissolving 0.656 g (7 mmol) of 2-methylimidazole and 7.52 g (0.2 mol) of ammonium hydroxide (NH_3_, 28–30% aqueous solution, Sigma-Aldrich, St. Louis, MO, USA). Solution A was added to solution B at room temperature while mixing. The resulting milky solution was mixed using a magnetic stirrer at room temperature for 1 h. The formed ZIF-8 product was separated from the supernatant by centrifugation at 4000 rpm for 10 min. Next, the product was washed by resuspension in 60 mL DI water under vigorous stirring to remove unreacted reactants and then separated by centrifugation, the supernatant was removed, and the remaining white powder was collected. The as-synthetized ZIF-8 products were dried in an electric oven at 60 °C for 12 h. The white powder of as-synthesized ZIF-8 is denoted as “ZIF.” Nearly one gram of product was derived after each synthesis.

Porous carbon was fabricated by pyrolysis of ZIF-8 in a tube furnace (Lindberg/Blue M Mini-Mite™, Waltham, MA, USA) with a tube size of 2.54 cm) at 800 °C with a heating rate of 10 °C min^−1^ and kept for 6 h under an argon atmosphere (argon 99.999%). The as-formed black powder (0.5 g) was washed with 37% hydrochloric acid, deionized water, and ethanol, respectively, and dried in a vacuum oven at 70 °C for 12 h. The sample is denoted as “CZIF.” The Se/CZIF composite was prepared using a melt diffusion method [27]. In a typical process, 300 mg sublimed selenium powder (−100 mesh; 99.5%, Sigma Aldrich, St. Louis, MO, USA) and 300 mg CZIF were mixed in a ratio of 1:1 to obtain a homogenous mixture and then sealed in a 50 mL stainless steel autoclave under argon gas. The mixture was then heated up to 260 °C for 12 h, resulting in 570 mg product. The capillary force was utilized via the melting diffusion technique to transfer Se into the pores of CZIF. Cathode electrodes were prepared by mixing Se/CZIF composite, carbon black (MTI Co., Richmond, BC, Canada), and sodium alginate (Ward’s Science Co., Ltd., Rochester, NY, USA) 0.5 wt% aqueous solutions) with a ratio of 8:1:1 to form a slurry. The resulting slurry was cast on an aluminum (Al) foil current collector using a doctor blade. The electrode was then vacuum dried at 60 °C overnight, then coated in a commercial ALD reactor (GEMStar^TM^ XT ALD System, Arradiance, MA, USA). Other types of Se/CZIF composites were also synthesized in previous studies [39,40,41].

The coating of alucone on the Se/CZIF electrode was performed at 100 °C by supplying trimethylaluminum (TMA) and glycerol to the MLD reactor. The source temperature for both trimethylaluminum (TMA) and GL was 25 °C. The precursor pipeline was maintained at 70 °C to avoid precursor condensation. Nitrogen gas (99.999%) was used as the carrier gas at a flow rate of 20 sccm. The base pressure in the ALD reactor was sustained at 200 miliTorr. The MLD procedure was set as follows: (1) a 2 s supply of TMA, (2) a 5 s extended exposure of TMA to the substrates, (3) a 10 s purge of oversupplied TMA and any by-products, (4) a 1 s supply of GL vapor, (5) a 5 s extended exposure of GL to the substrates, and (6) a 30 s purge of unreacted GL and any by-products. Using this reaction sequence, the thickness of the alucone deposited on the electrode was controlled by varying the number of MLD cycles. The thickness of the alucone on the Se/CZIF cathode was adjusted using 5, 10, and 20 MLD cycles (each cycle ~0.1 nm). The prepared samples were denoted as Se/CZIF-5 alucone, Se/CZIF-10 alucone, and Se/CZIF-20 alucone, respectively. The selenium loading of the electrode was calculated as 1.4 mg cm^−2^. Subsequently, the electrode was cut into round disks with a diameter of 12.7 mm for coin-cell assembly.

### 2.2. Structural Characterizations of ZIF, CZIF, and Se/CZIF Composite

The crystallinity and phase purity of the synthesized and modified ZIF-8-based samples and Se/CZIF composite were studied using X-ray diffraction (XRD). The X-ray diffraction patterns were collected using a Miniflex 600 6G (Rigaku, Tokyo, Japan) diffractometer (CuKα1, λ = 1.5406 nm, 40 kV, 15 mA,) in the 2θ range of 5° to 90° at a scan rate of 2°/min and a step size of 0.02 degree. The sample morphology was investigated using scanning electron microscopy (SEM-Philips XL30, Industrial and Electro-acoustic systems, Holland, The Netherlands) at an acceleration voltage of 20 kV. The samples were mounted on conductive double-sided sticky tape. A small coating of gold was applied to non-conductive ZIF samples to reduce the charging effects. Nitrogen adsorption–desorption isotherms were used to determine sample surface areas with a Quantachrome Autosorb-1 instrument (Boynton Beach, FL, USA) using the standard Brunauer-Emmett-Teller (BET) equation. The mesopore volume of each sample was estimated using a BJH plot, and the micropore volume was computed using the Lippens and de Boer t-plot technique using the adsorption data [36]. Before the BET analysis, all samples were degassed at 150 °C for 3 h under vacuum [36]. The thermal stability of the samples was studied using thermo-gravimetric analysis (TGA, TA instruments Discovery, New Castle, DE, USA) under an N_2_ atmosphere (5.0 purity, Linde Canada Inc. Praxair, Kelowna, BC, Canada) with a flow rate of 25 mL/min by heating the samples from 25 °C to 1000 °C at a heating rate of 10 °C/min.

### 2.3. Electrochemical Characterization of Se/C Cathode in Li-Se Batteries

CR2016 coin cells were used for electrochemical testing of the Se/CZIF composite. Coin cells were built in an argon-filled glovebox (H_2_O, O_2_ 0.1 ppm) using 1 M LiPF_6_ in ethylene carbonate/diethyl carbonate (1:1, *v*/*v*) with 3 vol.% FEC (abbreviated as LiPF_6_/EC-DEC + FEC), a Celgard 2400 (Celgard, Charlotte, NC, USA) separator, and a pure Li foil (Alfa Aesar, 99.9%) anode. Multi-channel battery testing equipment was used to perform galvanostatic charge/discharge in a voltage range of 1.0–3.0 V (vs. Li/Li^+^) at room temperature. Cyclic voltammetry (CV) and electrochemical impedance spectroscopy (EIS) characterizations were performed to investigate the electrochemical performance of cathodes in lithium-selenium batteries.

## 3. Results and Discussion

**Figure 1:**
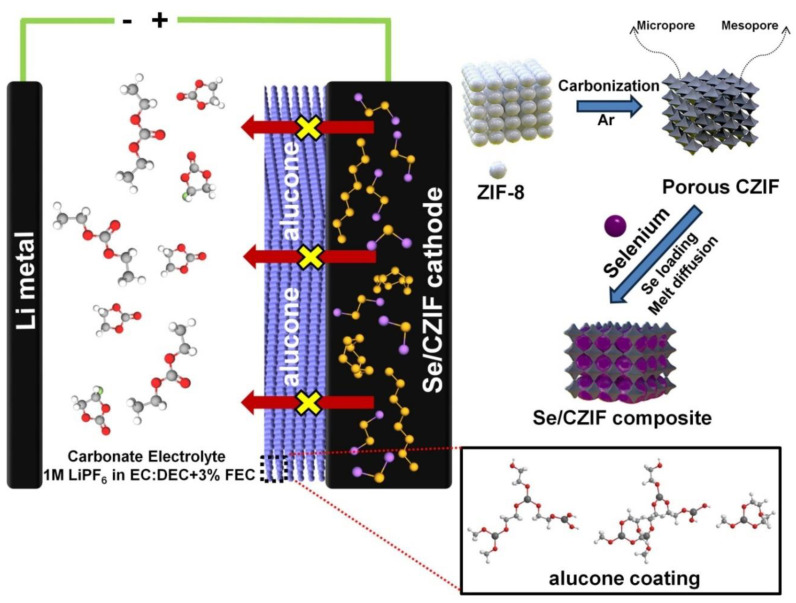
Schematic illustration for the preparation of the Se/CZIF−alucone coating.

Figure 1 demonstrates the steps for the preparation of the Se/CZIF-alucone-coated electrode. ZIF-8 white powder was carbonized under an argon atmosphere to prepare the porous carbon-containing micro/mesopores to confine active metal Se. The melting diffusion method was then applied to infiltrate Se into the carbon pores and obtain the Se/CZIF composite. The composite slurry was then pasted on an Al foil current collector. Finally, the ALD technique was used to coat an alucone thin film on the Se/CZIF electrode.

**Figure 2:**
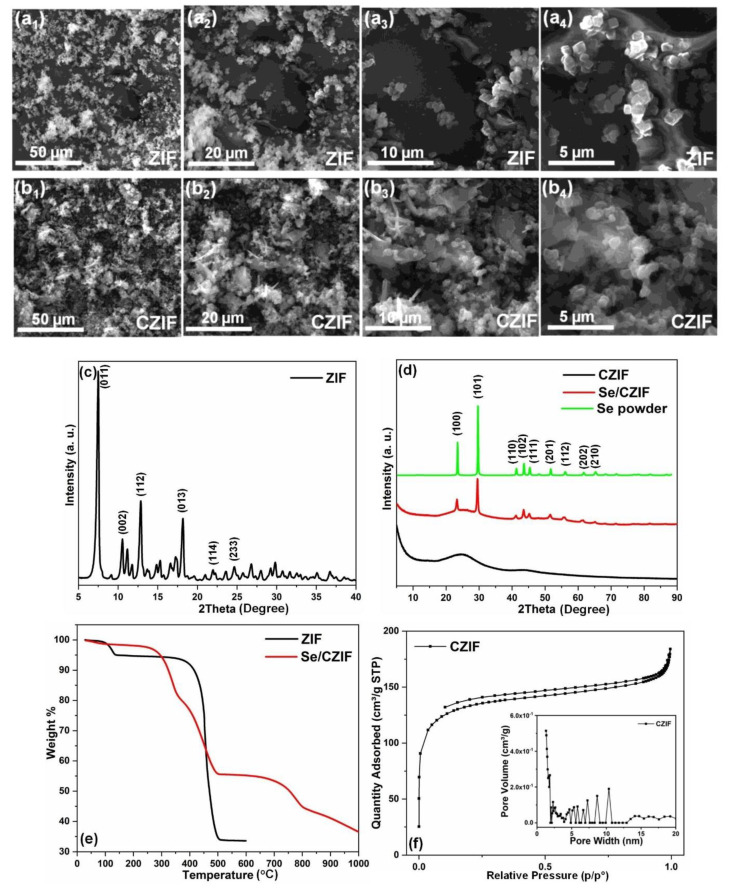
SEM images of (**a**) synthesized ZIF and (**b**) carbon derived from ZIF (CZIF) at different magnifications; XRD patterns of (**c**) synthesized ZIF and (**d**) CZIF, the Se/CZIF composite, and Se powder; (**e**) TGA spectrum of ZIF and Se/CZIF composite; and (**f**) N2 adsorption isotherm of CZIF (inset: pore size distribution of CZIF).

Figure 2a shows the SEM images of the as-synthesized ZIF-8 at different magnifications. The cubic/polyhedrol shapes of the ZIF-8 particles can be seen in these micrographs and correspond to the prepared ZIF-8 crystalline structure [36,42]. Figure 2b shows the SEM images of porous carbon derived from ZIF, named “CZIF” from here on out. The morphologies represent the irregular amorphous structure. The carbon pores confine Se and provide a facile pathway for the Li ion during the repeated lithiation–delithiation processes [43].

XRD analysis was performed on the as-synthesized ZIF-8 samples (Figure 2c). The ZIF-8 specimen exhibited sharp and high-intensity characteristic diffraction peaks at 7.50°, 10.52°, 12.86°, 14.84°, 16.62°, 22.28°, and 24.64°, which indicates the phase purity and formation of highly crystalline ZIF-8 products [36,42,44]. Figure 2d shows the XRD patterns of CZIF, the Se/CZIF composite, and Se. The XRD pattern of the CZIF sample showed only one broad peak at 25°, ascertaining the amorphous nature of CZIF. The Se/CZIF composite, however, revealed several sharp diffraction peaks at 23.4°, 29.6°, 41.2°, 43.6°, 45.5°, 51.6°, 56.2°, 61.6°, and 65.3°, confirming the incorporation of Se into the composite matrix. Furthermore, the residue infiltration was distributed as a bulk Se layer on the carbon matrix, contributing to the strong XRD diffraction peaks for Se/CZIF [27]. The distinct and sharp peaks of selenium powders have been demonstrated to compare the samples (JCPDS card No. 73-0465) [11,43].

Thermogravimetric analysis (TGA) of the ZIF sample is depicted in Figure 2e. The first weight loss of 5% at a temperature of about 130 °C could have been associated with the evaporation of adsorbed water and some other residual/absorbed/guest molecules from the cavities. There was no other detachable weight loss from 130 °C up to approximately 400 °C, indicating the thermal stability of the sample. A sharp weight loss of 60% occurred right after and was completed up to 500 °C. This can be attributed to the structural decomposition of ZIF-8 organic linkers (imidazolate) [38]. The TGA curve of the Se/CZIF composite (Figure 2e) showed a weight loss of about 55% between 300 to 500 °C, demonstrating the removal of Se from the carbon pores. The Se content of the carbon pores was estimated to be about 45 wt.%. At 700 °C, further weight loss was detected, which may have been due to the breakdown of the CZIF structure.

The N_2_ adsorption–desorption isothermal curves and corresponding pore size distribution of CZIF are displayed in Figure 2f. Before infiltration of selenium, the specific surface area of the CZIF was 428.8 m^2^ g^−1^, with a pore volume of 0.24 cm^3^ g^−1^. The pore size distribution data show highly uniform pores in the CZIF (pore diameter ~4.06 nm). The large surface area and pore volume of the carbon framework substantially impacted the selenium filling within the pores. The abundance of micropores allowed for the tight confinement of Se molecules within the porous carbon and the achievement of high Se loading, which is critical for sustained electrochemical performance.

**Figure 3:**
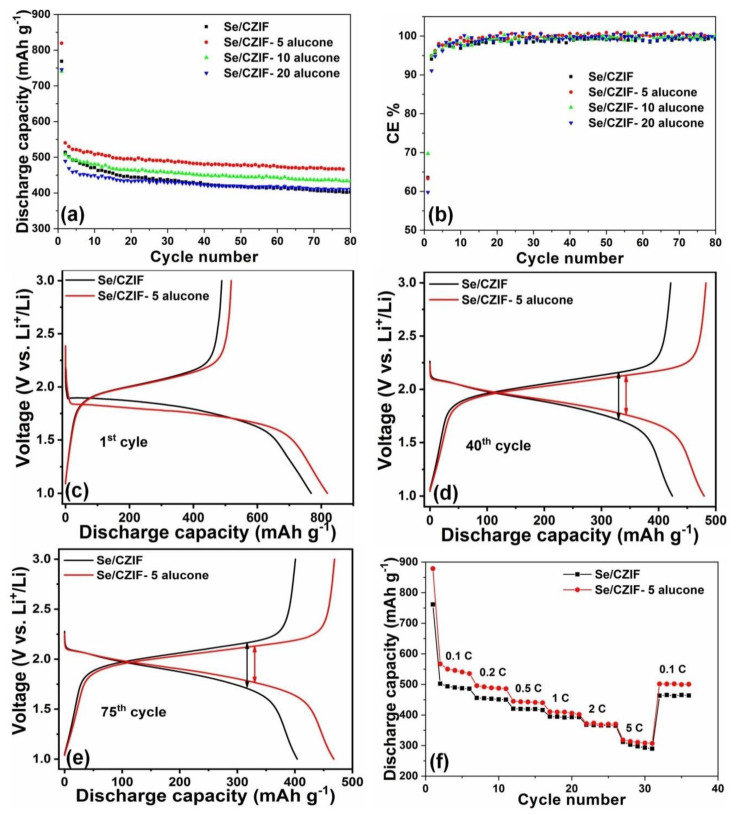
(**a**) Cycling performance and (**b**) Coulombic efficiency of Se/CZIF, Se/CZIF−5 alucone, Se/CZIF−10 alucone, and Se/CZIF−20 alucone measured at 0.1 C (1 C = 678 mA g^−1^); charge–discharge profiles of Se/CZIF and Se/CZIF−5 alucone in the (**c**) 1st cycle, (**d**) 40th cycle, and (**e**) 75th cycle; (**f**) rate capability of Se/CZIF and Se/CZIF−5 alucone.

The influence of the alucone coating on the electrochemical performance of the Se/CZIF cathode was studied in Li-Se batteries between 1 V and 3 V at 0.1 C. The results are shown in Figure 3. As shown in Figure 3a, Se/CZIF-5 alucone exhibited an initial discharge capacity of about 820 mAh g^−1^. In contrast, specific discharge capacities of 768, 740, and 745 mAh g^−1^ were delivered for pristine Se/CZIF, Se/CZIF-10 alucone, and Se/CZIF-20 alucone, respectively. This indicates that a ~0.5 nm thickness of alucone coating improves the Se/CZIF composite capacity performance. After the first cycle, the capacity dropped in all samples, likely due to solid electrolyte interface (SEI) film formation and the decomposition of electrolytes on the Se/CZIF cathode [45]. After the first cycle, the Se/CZIF-5 alucone retained the highest capacity among the samples after 80 cycles. For Se/CZIF, the capacity decreased gradually from 513 mAh g^−1^ in the second cycle to 402 mAh g^−1^ after 80 cycles. However, Se/CZIF-5 alucone delivered a specific capacity of 466 mAh g^−1^ after 80 cycles. Moreover, after 80 cycles, Se/CZIF-20 alucone exhibited a specific capacity lower than Se/CZIF-5 alucone and Se/CZIF-10 alucone but better than the pristine Se/CZIF cathode. The alucone coating was effective in enhancing the electrochemical properties of the Se/CZIF composite. Further increasing the alucone layer for more than 5 cycles (~0.5 nm) caused a noticeable decrease in the specific capacity [46,47,48,49]. This finding highlights the significance of alucone coating thickness in obtaining an optimum protective effect in Se cathodes. Except for the first cycle, the Coulombic efficiency was close to 100% for Se/CZIF-5 alucone, implying that the side reaction between the electrode and the electrolyte was reduced by an alucone layer (Figure 3b).

Figure 3c–e represents the charge/discharge curves of the Se/CZIF and Se/CZIF-5 alucone in the 1st, 40th, and 75th cycles tested at 0.1 C. Both Se/CZIF and Se/CZIF-5 alucone displayed one apparent plateau throughout the discharge and charge process, as shown in Figure 3c, which is typical of porous carbon-confined Se [40,45,50,51,52]. Moreover, it was found from Figure 3d,e that an alucone coating also minimized the polarization of the Se/CZIF cathode during the discharge and charge process. This indicates the enhancement in reaction kinetics of Se/CZIF by alucone coating. The enhanced capacity and kinetics of Se/CZIF-5 alucone may be attributed to the protective effect of nanoscale alucone covering, which alleviates polyselenide dissolution and reduces solid deposit polyselenides. Better rate capability was also observed in Se/CZIF-5 alucone, benefiting from the better kinetics with alucone coating. It can also be seen that Se/CZIF showed relatively better performance in terms of %degradation when the cell was tested at a higher C-rate of 2.0 and 5.0 C. When reaching the higher rates, the alucone coating could not entirely suppress the dissolution of polyselenide into the electrolyte because of a high ion exchange rate, resulting in losing its capability to protect the Se/CZIF composite (Figure 3f).

**Figure 4:**
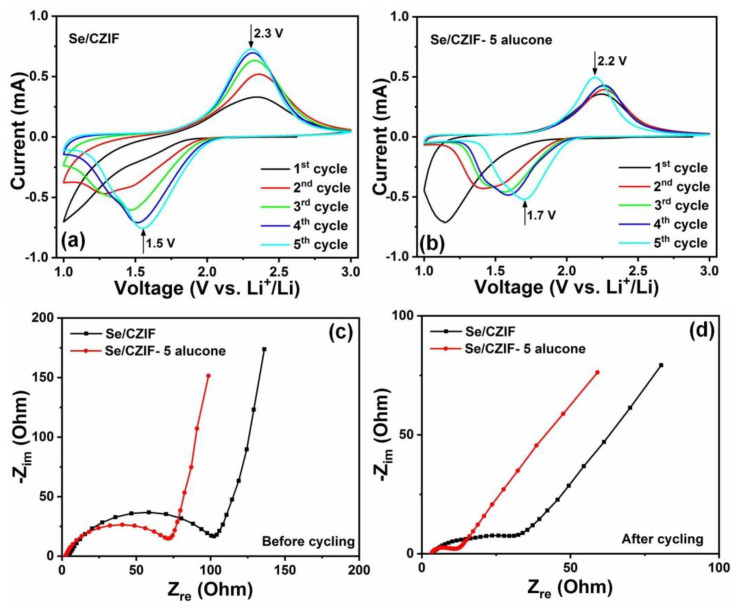
CV curves of the first 5 cycles for (**a**) Se/CZIF and (**b**) Se/CZIF−5 alucone, EIS curves of Se/CZIF and Se/CZIF−5 alucone for (**c**) before (**d**) after cycling.

CV analysis was conducted on the Se/CZIF and Se/CZIF-5 alucone cathodes at a scan rate of 0.2 mV s^−1^ to identify the causes for the enhanced performance by alucone coating, and the findings in the first five cycles are presented in Figure 4a,b. From the CV curves, it can be seen that for the Se/CZIF-5 alucone cathode, the oxidation and reduction peaks were relatively overlapped, indicating the enhanced reversibility of the Se/CZIF cathode by alucone coating. Additionally, with alucone coating, the spacing between the oxidation and reduction peaks of the Se/CZIF cathode was decreased from 0.8 V to 0.5 V in the fifth cycle, suggesting improved kinetics in the Se/CZIF-5 alucone cathode. For the Se/CZIF cathode, the reduction peaks showed a shoulder in all cycles, suggesting a multi-step phase transformation from Se to Li_2_Se. A pronounced decrease in the shoulder intensity can be seen in the Se/CZIF-5 alucone, offering a better kinetic and suppressive polyselenide dissolution with alucone coating. The comparison between Figure 4a,b suggests that the alucone coating effectively suppressed polyselenide dissolution from the Se/CZIF cathode into the carbonate electrolyte and the formation of a stable protecting layer on the Se/CZIF cathode via alucone coating. This hard coating layer on the cathode surface is critical for alloying and conversion electrodes, since the lithiation–delithiation process results in significant volume fluctuations.

To determine the process through which alucone coating improves performance, electrochemical impedance spectroscopy (EIS) measurement was performed on Se/CZIF and Se/CZIF-5 alucone before and after 100 cycles. The results are presented in Figure 4c,d. As seen in Figure 4c, both Se/CZIF and Se/CZIF-5 alucone demonstrated one semicircle in the high-frequency area, corresponding to the charge transfer resistance (R_CT_) at the electrode–electrolyte interface, and one inclined line in the low-frequency zone, corresponding to the finite length Warburg impedance [53]. The smaller R_CT_ in Se/CZIF-5 alucone may be attributed to the electrode material’s better physical contact with the Al current collector through an ultrathin alucone coating directly on the electrode, allowing for quicker electron diffusion [54]. The Nyquist profiles of Se/CZIF after cycling (Figure 4d) presented one semicircle (R_CT_) in the high-frequency region, Warburg impedance in the low-frequency domain, and another semicircle in the medium-frequency region, which can be assigned to the Li ion diffusion resistance at the SEI (R_SEI_). From the result, we can see that for the Se/CZIF-5 alucone cathode, R_SEI_ and R_CT_ decreased. EIS research clearly shows that coating the Se/CZIF cathode with alucone improves the development of SEI layers and decreases charge transfer and Li ion diffusion resistances [55]. The Nyquist profiles were fitted by using the equivalent circuit to obtain EIS parameters (Table 1). Therefore, the lithiated alucone in our work might have contributed to the reduced R_CT_ and R_SEI_ on Se/CZIF-5 alucone compared to Se/CZIF.

**Figure 5:**
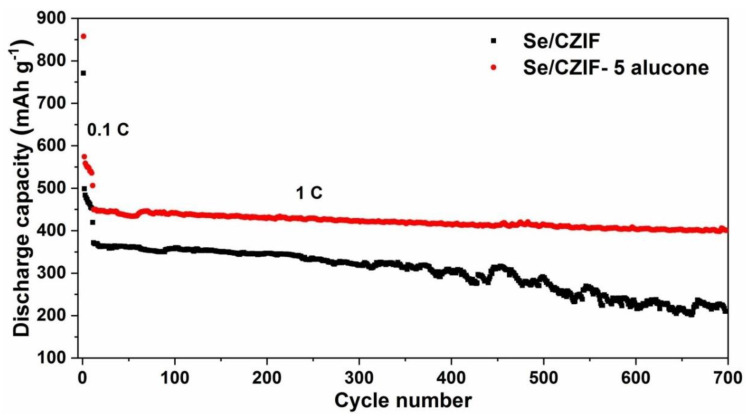
Long cycling performance of Se/CZIF and Se/CZIF−5 alucone at a rate of 0.1 C during the first 10 cycles and 1 C for the subsequent cycles.

Finally, after 700 cycles of cycling at 1 C, Se/CZIF-5 alucone may retain a specific capacity of 400 mAh g^−1^ (Figure 5). Based on the findings, it is possible to infer that the alucone coating enhanced the specific capacity, cycle stability, and rate capability of the Se/CZIF cathode in Li-Se batteries.

## 4. Conclusions

A novel porous carbon/Se cathode was developed using zeolitic imidazolate framework-8 (ZIF-8)—a subclass of MOFs—as the precursor for porous carbon to build an Li-Se battery. The advancement of the electrode–electrolyte interface using MLD alucone coating worked as a novel and potential universal approach to enable high-performance Li-Se batteries with conventional Se/C electrodes. ZIF-8-derived porous carbon exhibited high porosity, conductivity, large surface area, and good chemical stability, resulting in improved lithium storage performance in the Se/C electrodes. The Se/CZIF-5 alucone electrode demonstrated a stabilized ultra-long cycle life with a capacity of over 400 mAh g^−1^ after 700 cycles at 1 C, representing very stable high-temperature Li-Se batteries with prolonged cycle performance. The utilization of MLD enables the usage of conventional Se/C cathode materials for Li-Se batteries with carbonate electrolytes. It is a facile and versatile approach that applies to various Se/C electrodes without redesigning the carbon host materials.

## Figures and Tables

**Table 1:** EIS parameters obtained by fitting the Nyquist plots of Se/CZIF and Se/CZIF-5 alucone after 100 cycles.

	Before Cycling		After 100 Cycles		
	R_i_ (Ω)	R_CT_ (Ω)	R_i_ (Ω)	R_CT_ (Ω)	R_SEI_ (Ω)
Se/CZIF	3.6	85.7	3.7	4.7	16.0
Se/CZIF-5 alucone	2.2	52.4	3.3	2.5	6.7
